# Paediatric palliative care: development and pilot study of a ‘Directory’ of life-limiting conditions

**DOI:** 10.1186/1472-684X-12-43

**Published:** 2013-12-11

**Authors:** Richard Hain, Mary Devins, Richard Hastings, Jayne Noyes

**Affiliations:** 1 Paediatric Palliative Medicine, Children’s Hospital, Heath Park, Cardiff CF14 4XN, UK; 2 School for Healthcare Sciences, Bangor University, Bangor, LL57 2EF, UK; 3 University of South Wales, Heath Park, Cardiff CF14 4XN, UK; 4 Paediatric Palliative Medicine, Our Lady’s Children’s Hospital and The Coombe Women’s Hospital, Dublin, Republic of Ireland; 5 School of Psychology, Bangor University, Bangor LL57 2EF, UK

**Keywords:** Palliative care, Symptom control, Epidemiology, Trajectory, Public health

## Abstract

**Background:**

Children’s palliative care services are developing. Rational service development requires sound epidemiological data that are difficult to obtain owing to ambiguity in the definitions both of the population who needs palliative care and of palliative care itself. Existing definitions are of trajectory archetypes. The aim of this study was to develop and pilot a directory of the commonest specific diagnoses that map on to those archetypes.

**Methods:**

The diagnoses of patients under the care of five children hospices and a tertiary specialist palliative medicine service in the UK were recorded. Duplicates and diagnoses that were not life-limiting conditions according to the ACT/RCPCH criteria or were not primary were removed. The resulting Directory of life-limiting conditions was piloted by analysing Death Certificate data of children in Wales between 2002 and 2007.

**Results:**

1590 diagnoses from children’s hospices and 105 from specialist palliative medicine were combined. After removals there were 376 diagnostic label. All ICD10 chapter headings were represented by at least one condition. The pilot study showed that 569 (54%) deaths in Wales were caused by LLC. Only four LLC resulted in ten or more deaths. Among deaths from LLC, the ten commonest diagnoses accounted for 32%, while the 136 diagnoses that caused one or two deaths accounted for 25%. The majority occurred from a small number of life-limiting conditions.

**Conclusion:**

The Directory is a practical tool for identifying most life-limiting conditions using ICD10 codes that facilitates extraction and analysis of data from existing sources in respect of life-limiting conditions in children such as death certificate data, offering the potential for rapid and precise studies in paediatric palliative care.

## Background

The need of children for palliative care is well recognised [1-9] but difficult to define. It is defined by the needs of an individual child and family when cure is no longer possible, rather than by age or organ system. The Royal College of Paediatrics and Child Health (RCPCH), working with the Association for Children’s Palliative Care (ACT) in 1997, defined the concept of life-limiting condition [6] through a series of archetype descriptions (Table 1), but did not attempt to name specific diagnoses except as exemplars. If, however, children are to have the same access to specialist palliative interventions as adults currently enjoy, service developers must engage commissioners. That requires a precise understanding of the numbers of children who need services, which in turn requires specific diagnostic criteria.

**Table 1:** **ACT/RCPCH Categories**[6]

**Category**	**Key characteristic**	**Description**	**Examples**
I	**Potential for cure** - life is threatened, not necessarily limited.	Conditions for which treatment may be feasible but can fail	Cancer
			Some cardiac anomalies
II	**Period of normality** despite having fatal diagnosis.	Conditions where premature death is inevitable but where there may be long periods of participation in normal activities	Duchenne Muscular Dystrophy
III	**Relentless deterioration** from, or before, time of diagnosis.	Progressive conditions without curative treatment options, where treatment is exclusively palliative and commonly extends over many years	Metabolic or neurodegenerative conditions
IV	**Unpredictable** course whose progression is not easily judged from natural history.	Irreversible but non-progressive conditions causing likelihood of premature death through complications	Severe cerebral palsy
			Traumatic brain injury
			Septic brain injury

We developed a Directory of life-limiting conditions by mapping the four ACT/RCPCH archetypes onto the diagnoses of actual patients admitted to hospice or palliative care services in the UK. We then piloted the Directory by using it to interrogate death certificate data for children in Wales over a five-year period.

We describe development of the Directory and, in the light of results of the pilot study, consider some of its current limitations as well as the wider applications in taking forward service and research developments in children’s palliative care. The aim of this study was to develop and pilot a tool that can largely define the group of conditions that are life-limiting in childhood and facilitate secondary analysis of epidemiological data in this under-researched field.

## Methods

### Raw diagnostic data were collected

Diagnoses were obtained from patients under the care of five children’s hospices that were using a standardised data collection tool developed by Chase Hospice (Esplen, personal communication 2010), and the Welsh specialist paediatric palliative medicine service based at the Children’s Hospital in Cardiff. All had been considered to be ‘life-limiting’ both by the referring clinician and the clinician accepting the referral.

### The list of diagnostic labels was refined

The list was edited in three ways.

Removal of duplicate diagnoses.

Duplicates occurred when two or more terms were used to describe the same condition (e.g., trisomy 13 and Patau’s syndrome).

Removal of non-diagnoses.

This included terms that had led to referral, but were not life limiting conditions in themselves. They included *modes of death* (e.g., apnoea), *treatments* for the life limiting diagnosis (e.g., tracheostomy) and conditions that were *incidental* to the life-limiting diagnosis (e.g., anaemia).

Removal of diagnoses that were not life-limiting.

For the purposes of this study, a life-limiting diagnosis was considered to be any condition whose trajectory could be described by one or more of the ACT/RCPCH archetypes (Table 1).

### ICD10 codes were assigned to each diagnosis

A diagnostic label and code from the International Classification of Disease (ICD10) was assigned by the investigators to each diagnosis on the list (apps.who.int/classifications/apps/icd/icd10online/).

### The draft directory was piloted using Welsh death certificate data

The draft Directory was used to interrogate a database comprising aggregated anonymous death certificate data for all deaths in Wales between 0 and 19 years between 2002 and 2007, obtained from Public Health Wales Observatory [10]. LLC that mapped onto one or more of the ACT/RCPCH archetypes but did not already appear in the draft Directory, were added to the draft.

This was a secondary analysis of data that were anonymous or already in the public domain that formed part of the My Choices project. Ethical approval for the project was obtained from the Betsi Cadwaldr NHS Research Ethics Committee.

## Results

### Development

1590 diagnoses from children’s hospices and 105 from specialist palliative medicine were combined. 1319 diagnoses were removed (see Methods section). All ICD10 chapter headings were represented by at least one condition, showing the range of conditions that can limit life in children.

### Pilot study

There were 1052 deaths in childhood in Wales between 2002 and 2007 (Tables 1, 2 and 3). Of these, 569 (54%) were caused by LLC according to the Directory. Of 382 diagnoses listed causes of death on certificates, 186 (49%) were not LLC according to the Directory.

**Table 2:** Top five causes of death

**Number of deaths**	**Diagnosis**	**ICD10 code**
26	Cerebral palsy, unspecified	G80.9
22	Malignant neoplasm: Brainstem, brain (unspecified)	C71.7, C71.9
15	Acute leukaemia (lymphoblastic, myeloid)	C91.0, C92.0
11	Epilepsy, unspecified	G40.9
9	Muscular dystrophy	G71.0

These are causes of death that are describable by one of more of the ACT/RCPCH categories and therefore considered ‘life-limiting’ conditions.

**Table 3:** Top eleven diagnoses accounting for three or more deaths among neonates

**Number of deaths**	**Diagnosis**	**ICD10 code**
60	Birth asphyxia, unspecified	P21.9
19	Hypoplasia and dysplasia of lung	Q33.6
16	Necrotising enterocolitis of fetus and newborn	P77
16	Congenital diaphragmatic hernia	Q79.0
10	Edwards’ syndrome, unspecified	Q91.3
8	Congenital malformation of heart, unspecified	Q24.9
7	Persistent fetal circulation	P29.3
4	Congenital renal failure	P96.0
4	Hypoplastic left heart syndrome	Q23.4
3	Severe birth asphyxia	P21.0
3	Patau’s syndrome, unspecified	Q91.7

These are causes of death describable by one of more of the ACT/RCPCH categories and therefore considered ‘life-limiting’ conditions. In total, there were 169 deaths from 24 diagnoses.

According to the Directory, Death Certificate data recorded 169 deaths from life-limiting conditions (Table 3). 97% of which were accounted for by conditions in only two ICD10 chapters. Only four LLC resulted in ten or more deaths (Table 2). Among deaths from LLC, the ten commonest diagnoses accounted for 32%, while the 136 diagnoses that caused one or two deaths accounted for 25%. The majority occurred from a small number of life-limiting conditions. Malignancy (25%) and neurological conditions (21%) were the most frequent.

## Discussion

Defining the population of children with life-limiting conditions accurately requires precise diagnoses. The aim of this study was to develop, and then to pilot, a list of life-limiting diagnoses in children that can be used for immediate secondary analysis of existing data.

In children, the term ‘life-limiting condition’ encompasses non-malignant as well as malignant conditions and the range of conditions is wide. LLC in children, especially in the UK, are conventionally classified by the ACT/RCPCH system [2,5,7,9], which relies for its validity on assumed commonality among the courses of diseases within each of four categories. Limited evidence [11] supports this concept, but the ACT/RCPCH categories as they stand are too vague to be effective as registration criteria and need to be supplemented by identifying precise diagnoses. We are not, of course, the first to recognise the need for specific data in service development. Lists of life-limiting conditions have been compiled before, notably by Knapp (personal communication 2011), Craig [9] and Feudtner [12,13].

The virtue of the ACT/RCPCH system is that it captures the diversity of conditions that can limit life; our aim was to obtain useful precise data without losing that virtue. For the purposes of this study, a life-limiting condition is therefore a condition whose trajectory is plausibly described by one or more of the ACT/RCPCH archetypes.

Diagnoses that emanated from hospices were not the same as those from specialist PPM services. Children’s hospices typically offer short respite stays and are often nurse-led. In contrast, specialist PPM services are based around availability of specialist medical services. Although the two populations clearly significantly overlap, they are not precisely co-terminous [14], and combining them therefore further expanded the number of diagnoses on the list.

It could be argued that some individual children with diagnoses that are not life-limiting conditions nevertheless require care that is, in effect, palliative. Traffic injuries [15], for example, do not fit an ACT/RCPCH category. For children with severe injuries that lead to death, however, PPM services could have a valuable role such as supporting end-of-life discussions in intensive care. Perhaps this indicates a potential value in extending the ACT/RCPCH categories to reflect the broader role that might be played by PPM services.

Conditions such as diabetes and epilepsy are usually incurable and often require the same approach and ‘ethos’ as palliative care. There is certainly a risk of drawing an arbitrary distinction between palliative care and what is simply good clinical practice in children. Nevertheless, those working in the field recognise a population of children within this wider group who are at high risk of death during childhood, and in whom complex symptom control is a frequent clinical challenge. It is that population that the Directory aims to help to identify.

There are inevitable limitations to a study of this nature. The ACT/RCPCH archetypes provide a measure of objectivity but still rely on a certain degree of subjective judgment. It is not possible to list every possible LLC in a Directory: the pilot study enabled us to add some diagnostic labels that might otherwise have been missed, but if the Directory is to remain current there will need to be a mechanism for adding new diagnoses as they become apparent.

Publically available data recorded on death certificates is limited to the principal causes of death. It is possible that small numbers of children with LLC who died from incidental causes were not identified in our pilot study.

In interpreting these results, it is important to make a distinction between diagnoses and patients. The Directory lists diagnoses. While it would not be valid to draw conclusions about number of children needing access to palliative care solely from observations of the number who actually do so, observations of service use do provide a valid source of *diagnoses,* since it is extremely likely (though not certain) that every important LLC would occur at least once. Similarly, the Directory is designed simply to provide a tool for analysing epidemiological data. It would be impossible to draw conclusions about the numbers of children suffering from life-limiting conditions from the Directory alone. Effective use of the Directory relies on applying it to databases that include accurate and detailed recording of ICD10 diagnostic labels to subheading level.

On the other hand, the Directory was easy to use and enabled the authors to interrogate a robust existing database effectively and immediately. We were able to make some important observations about LLC as causes of death in in Wales over a reasonable study period of five years. Most individual LLC caused only one death over that period and very few diagnoses (5 in neonates, 7 in older children) caused it 10 times or more (Tables 1 and 2). At the same time, nearly one third of deaths were accounted for by only ten different LLC, confirming clinicians’ impression that, while the range of possible LLC is wide, it is possible to identify a relatively small number of diagnoses whose symptom management should form the core of a specialist palliative care skillset. Of 420 deaths from LLC outside the neonatal period, 75% were from conditions other than cancer. This is higher than in studies that have relied on reporting by paediatricians [14], suggesting there has been under-recognition of the life-limiting nature of non-malignant conditions.

The Directory has a number of immediate practical applications where the sub-population of children with LLC needs to be identified within larger groups such as those with complex chronic disability or other chronic illness. It can rationally underpin fair admission and referral criteria for children’s hospice services, and help evaluate the magnitude of the need for specialist palliative medicine and palliative care services for children by institutions within the National Health Service. In countries such as the USA with a private healthcare system, the Directory can inform funding decisions among insurance companies. It can also facilitate robust governance and record-keeping by those providing palliative care, by allowing a definition of palliative care derived from a standard that has been largely agreed.

The Directory has potentially important applications for research in paediatric palliative care. To define the population of children needing palliative care in an essential first step in considering any research question that impacts specially on that group. The Directory has already been used for this purpose in research [16,17] and service development [18]. Prevalence data, in particular, are key to rational service development, but for LLC there is no consistent relationship with incidence. Given the long natural history of LLC [11], it is usually impractical to obtain the prospective data needed to establish prevalence. The pilot study of the Directory shows that an agreed list of diagnoses potentially allows immediate secondary analysis of existing data.

Finally, the Directory can potentially allow critical evaluation of the ACT/RCPCH categories themselves, allowing amendments and improvements to what has become the standard definition of what constitutes a ‘life-limiting condition’.

## Conclusions

The authors have compiled a ‘Directory’ of ICD10 diagnoses, drawing on admissions to children’s hospices on the one hand, and referrals to specialist paediatric palliative medicine on the other.

A pilot study of the Directory to analyse death certificate data showed that it was easy to use and allowed immediate secondary analysis of an established database. The study showed that around half of all childhood deaths in the study period were from LLC, thje majority of LLC are non-malignant, and that the range of LLC causing death in the neonatal period was markedly narrower than outside it.

By defining a list of precise ICD10 codes that map onto ACT/RCPCH criteria, for the first time the Directory allows analysis of existing clinical databases, paving the way for rapid establishment of prevalence data that would otherwise have been impractically slow.

No list of LLC based on disease label can ever be exhaustive. As new diagnoses become apparent, expansion will be important and should be the basis for further studies, which should also attach Read and ACT/RCPCH categories. While it will therefore continue to need refinement, the Directory is a key tool for rational service development in children’s palliative care.

## Competing interests

The authors declare that they have no competing interests.

## Authors’ contributions

RH conceived of the study, supervised the data collection and wrote the manuscript. MD carried out the data collection. RH, RHastings, MD and JN all developed the Directory itself, making amendments in various iterations. All authors participated in development of the final manuscript and have seen and approved the submitted draft.

## Pre-publication history

The pre-publication history for this paper can be accessed here:

http://www.biomedcentral.com/1472-684X/12/43/prepub

## References

[B1] ACT The Transition Care Pathway A Framework for the Development of Integrated Multi-Agency Care Pathways for Young People with Life-threatening and Life-limiting Conditions 2007 Bristol, UK: Together for Short Lives

[B2] ACT A Guide to the Development of Children’s Palliative Care Services 2009 3 Bristol, UK: Together for Short Lives

[B3] ACT A Neonatal Pathway for Babies with Palliative Care Needs 2009 Bristol: ACT

[B4] Katrina McNamara-Goodger LM ACT Right People, Right Place, Right Time: Planning and developing an effective and responsive workforce for children’s and young people’s palliative care 2009 Bristol: ACT

[B5] ACT, McNamara-Goodger) LCaK A Family Companion to the ACT Care Pathway for children with life-limiting and life-threatening conditions 2009 Bristol: ACT

[B6] ACT/RCPCH A guide to the development of children’s palliative care services 1997 1 Bristol and London: ACT/RCPCH

[B7] ACT/RCPCH A Guide to the Development of Children’s Palliative Care Services - Second Edition, September 2003 2003 Bristol, UK: Together for Short Lives Updated report. ACT/RCPCH

[B8] Brook L Hain R Predicting death in children Arch Dis Child 2008 12 12 1067 1070 10.1136/adc.2007.127332 18562454

[B9] Craft PSA Killen S Palliative care services for children and young people in England 2007 London: Department of Health

[B10] Public Health Wales Observatory Available from: http://www.publichealthwalesobservatory.wales.nhs.uk/. (accessed September 2011)

[B11] Wood F Simpson S Barnes E Hain R Disease trajectories and ACT/RCPCH categories in paediatric palliative care Palliat Med 2010 12 8 11 Epub 2010/08/20 10.1177/0269216310376555 20719816

[B12] Feudtner C Hays RM Haynes G Geyer JR Neff JM Koepsell TD Deaths attributed to pediatric complex chronic conditions: national trends and implications for supportive care services Pediatrics 2001 12 6 E99 Epub 2001/06/05 10.1542/peds.107.6.e99 11389297

[B13] Feudtner C Kang TI Hexem KR Friedrichsdorf SJ Osenga K Siden H Pediatric palliative care patients: a prospective multicenter cohort study Pediatrics 2011 12 6 1094 1101 Epub 2011/05/11 10.1542/peds.2010-3225 21555495

[B14] Hain RDW Palliative care in children in Wales: a study of provision and need Palliat Med 2005 12 137 142 10.1191/0269216305pm967oa 15810753

[B15] Pearson GA Ward-Platt M Kelly D How children die: classifying child deaths Arch Dis Child 2011 12 10 922 926 Epub 2010/07/27 10.1136/adc.2009.177089 20656738

[B16] Fraser LK Miller M Hain R Norman P Aldridge J McKinney PA Rising national prevalence of life-limiting conditions in children in England Pediatrics 2012 Epub 2012/03/14 10.1542/peds.2011-2846 22412035

[B17] Totsika V Noyes J Hastings R Hain R Report on DicData group of Projects (supplementary/final report) 2013 Bristol, UK: Together for Short Lives

[B18] Cheung R CHIMAT Atlas of Variation 2011 London: Department of Health [cited 2011 September]; Available from: http://www.chimat.org.uk/tools/atlasofvariation

